# Supplementary material for: On the path to reference genomes for all biodiversity: laboratory protocols and lessons learned from processing over 2,000 species in the Sanger Tree of Life
Source: Gigascience. 2025 Oct 23;14:giaf119. doi: 10.1093/gigascience/giaf119 (PMC12548527; doi:10.1093/gigascience/giaf119)
Supplement: giaf119_Authors_Response_To_Reviewer_Comments_Original_Submission [file giaf119_authors_response_to_reviewer_comments_original_submission.pdf]

Author's Response To Reviewer Comments

Reviewer 1

1. Sample processing:
(1) Sampling of rare and endangered species: for such a large-scale study of the "Tree of Life", it is bound to involve some species that are difficult to obtain conventional tissues, therefore the manuscript may include a section on how to select suitable tissues for subsequent experiments, especially for rare species. And is it possible to provide a prioritized list of tissues selection based on the difficulty of extracting high-quality DNA?

Our main experience in this area is restricted to chordates, additional text has been added to make this more clear (pg 22) and to prioritise tissues (pg 23).

(2) Processing and extraction of unconventional tissues: accordingly, it is recommended to add content regarding sample processing and extraction procedures for unconventional tissues, e.g., any particular methods to improve the quality of DNA extraction.

The samples received with ToL are provided by our network of collaborators, who have worked hard to comply with our submission requirements. Due to this we have relatively little experience of unconventional tissue types.
Somewhat unconventional materials and methods are usually clade specific and therefore already included in the provided descriptions (e.g. corals and porifera)

(3) Sample contamination problem is often overlooked yet critical: how to reduce sample contamination problems in large-scale sample processing and other experimental processes? How to exclude sample or experimental contamination from data?

The avoidance of DNA amplification in processes reduces the impact of contamination significantly, and this is therefore not an issue for the majority of ToL samples. Any non-target sequences are separated bioinformatically and text has been added to describe this.
Samples that require a ULI or amplification-based approach, such as protists, are impacted much more by this issue, but again the solution here is to decontaminate the data, not the sample. Text has been added to this section to emphasize this (pg 25). Physical separation approaches, such as filtering for protist samples, have been trialled but were not successful.

2. Analyzing method limitations: while the manuscript mentions some challenges that may be encountered in the processing of samples from various taxa, there is little discussion on the limitations of those experimental methods. It is recommended to expand the content of the limitations of the methods, such as some methods may not work well for certain types of samples, or some steps may have factors that affect the accuracy of the results, so that readers can have a more comprehensive understanding of the scope of application and potential problems of the method.

The limitations of the methods are shown by the pass/fail metrics for the overall process, and for each taxonomic group. High failure rates indicate that a method is not producing the required results for progression. Further, CCS yields that fall below the thresholds show limited data yield. Where methods are limited in a particular clade, this is described in the section and our best alternative method provided.

When methods fail, we do not have clear assays to understand the reason behind the failure, and we resort to trial and error. This is a limitation of all methods and as far as we know, there are no assays to evaluate why material that has been preserved and stored properly fails to give good results.

3. The manuscript is currently organized according to the experimental procedures, but some of the more relevant components could probably be consolidated to reduce redundant information and improve the readability. The authors studied the experimental conditions for different taxa in long read sequencing and Hi-C library preparation, but fail to emphasize their relevance in the introduction.

We are happy to remove redundant sections and improve streamlining but would benefit from more specific suggestions if possible, as we have tried to do this already.

Text has been added to emphasise the importance of long read and HiC data for genome assembly (pg 2)

Reviewer 2

One thing that might be changed is the title. From first reading it I expected to hear also about assembly strategies, as well as some comparisons and oddities of the yielded genomes. It is great to have the manuscript as it is, but I like to see it better reflected in the title that the main focus here is on the wet lab part, especially the extraction of good quality DNA/RNA.

The title has been altered with the aim of emphasising the laboratory focus of the paper.

I have some issues with the figures:
Fig. 7: there is no mention in the legend about the y-axis scale - I assume from the text that it refers to Gigabases?
The figure legend has been altered to include the unit of measure.
Figs. 8,9, 11-15: It is a bit confusing until I realised the log scale of the numbers. I would prefer to see it not with a log scale, but in a similar way as Fig. 6, with percentages on display, and an accompanying species number somewhere on the side. In the way it is shown now, the failed proportion looks so small and gives a wrong impression. Maybe overthink the colors, I would prefer another color for the Pass ULI, which is more similar in tone with Pass, because at the moment pass ULI and fail are similar in tone and brightness and appear as being opposed to the green "pass", while the difference between "fail" and the rest should be more pronounced in my view.

The logarithmic scale has been changed to a % scale with the total number of species included added on the side. The colours have been altered to an accessible colour format and the ‘Pass’ and ‘Pass ULI’ categories are now more similar to one another in colour than the ‘Fail’.

em Editorial Manager®

Cookie Preference Center

We use cookies which are necessary to make our site work. We may also use additional cookies to analyze, improve and personalize our content and your digital experience. For more information, see our Cookie Policy.

You may choose not to allow some types of cookies. However, blocking some types may impact your experience of our site and the services we are able to offer. See the different category headings below to find out more or change your settings.

Allow all

Manage Consent Preferences

Strictly Necessary Cookies

Always active

These cookies are necessary for the website to function and cannot be switched off in our systems. They are usually only set in response to actions made by you which amount to a request for services, such as setting your privacy preferences, logging in or filling in forms. You can set your browser to block or alert you about these cookies, but some parts of the site will not then work.

Functional Cookies

These cookies enable the website to provide enhanced functionality and personalisation. They may be set by us or by third party providers whose services we have added to our pages. If you do not allow these cookies then some or all of these services may not function properly.

Performance Cookies

These cookies allow us to count visits and traffic sources so we can measure and improve the performance of our site. They help us to know which pages are the most and least popular and see how visitors move around the site.

Targeting Cookies

These cookies may be set through our site by our advertising partners. They may be used by those companies to build a profile of your interests and show you relevant adverts on other sites. If you do not allow these cookies, you will experience less targeted advertising.

Cookie List

Clear

Apply Cancel

Consent Leg.Interest

Confirm my choices

Powered by onetrust
