# Supplementary material for: On the path to reference genomes for all biodiversity: laboratory protocols and lessons learned from processing over 2,000 species in the Sanger Tree of Life
Source: Gigascience. 2025 Oct 23;14:giaf119. doi: 10.1093/gigascience/giaf119 (PMC12548527; doi:10.1093/gigascience/giaf119)
Supplement: giaf119_Reviewer_2_Report_Original_Submission — Lars Podsiadlowski -- 5/28/2025 [file giaf119_reviewer_2_report_original_submission.pdf]

Reviewer Report

Title: On the path to reference genomes for all biodiversity: laboratory protocols and lessons learned from processing over 2000 species in the Sanger Tree of Life

Version: Original Submission **Date:** 5/28/2025

Reviewer name: Lars Podsiadlowski

Reviewer Comments to Author:

The Authors provide a profound overview over their aim to generate genome information for a wide range of species in the tree of life project. As a scientist with hands on experience on genome sequencing, I greatly appreciated all the information here, especially detail on the differences experienced with different taxa, as this is probably the most important lesson here, that there is high variation and strategies must be adapted to that. I am also happy that many of the approaches are also available as detailed online protocols, which really helps a lot in practical work. The selected examples of size profiles also give a good impression on what differences can be expected, e.g. with different extraction methods applied to the same species. Although detailed, I think that the authors provide a lot of relevant information here and would not change that. I did also not spot any errors or flaws in the text.One thing that might be changed is the title. From first reading it I expected to hear also about assembly strategies, as well as some comparisons and oddities of the yielded genomes. It is great to have the manuscript as it is, but I like to see it better reflected in the title that the main focus here is on the wet lab part, especially the extraction of good quality DNA/RNA.I have some issues with the figures:Fig. 7: there is no mention in the legend about the y-axis scale - I assume from the text that it refers to Gigabases?Figs. 8,9, 11-15: It is a bit confusing until I realised the log scale of the numbers. I would prefer to see it not with a log scale, but in a similar way as Fig. 6, with percentages on display, and an accompanying species number somewhere on the side. In the way it is shown now, the failed proportion looks so small and gives a wrong impression. Maybe overthink the colors, I would prefer another color for the Pass ULI, which is more similar in tone with Pass, because at the moment pass ULI and fail are similar in tone and brightness and appear as being opposed to the green "pass", while the difference between "fail" and the rest should be more pronounced in my view.

Level of Interest

Please indicate how interesting you found the manuscript: Choose an item.

Quality of Written English

Please indicate the quality of language in the manuscript: Choose an item.

Declaration of Competing Interests

Please complete a declaration of competing interests, considering the following questions:

- Have you in the past five years received reimbursements, fees, funding, or salary from an organisation that may in any way gain or lose financially from the publication of this manuscript, either now or in the future?
- Do you hold any stocks or shares in an organisation that may in any way gain or lose financially from the publication of this manuscript, either now or in the future?
- Do you hold or are you currently applying for any patents relating to the content of the manuscript?
- Have you received reimbursements, fees, funding, or salary from an organization that holds or has applied for patents relating to the content of the manuscript?
- Do you have any other financial competing interests?
- Do you have any non-financial competing interests in relation to this paper?

If you can answer no to all of the above, write 'I declare that I have no competing interests' below. If your reply is yes to any, please give details below.

I declare that I have no competing interests

I agree to the open peer review policy of the journal. I understand that my name will be included on my report to the authors and, if the manuscript is accepted for publication, my named report including any attachments I upload will be posted on the website along with the authors' responses. I agree for my report to be made available under an Open Access Creative Commons CC-BY license (http://creativecommons.org/licenses/by/4.0/). I understand that any comments which I do not wish to be included in my named report can be included as confidential comments to the editors, which will not be published.

Choose an item.

To further support our reviewers, we have joined with Publons, where you can gain additional credit to further highlight your hard work (see: https://publons.com/journal/530/gigascience). On publication of this paper, your review will be automatically added to Publons, you can then choose whether or not to claim your Publons credit. I understand this statement.

Yes Choose an item.
